# The Burden of Surgical Cancellations: A Quality Improvement Study on the Importance of Preoperative Assessment

**DOI:** 10.7759/cureus.21731

**Published:** 2022-01-30

**Authors:** Saphalya Pattnaik, Sheetal K Dixit, Vandana Bishnoi

**Affiliations:** 1 Trauma and Orthopaedics, University Hospital Lewisham, Lewisham, GBR; 2 Quality Improvement, Dr. D. Y. Patil Medical College, Hospital & Research Centre, Pune, IND

**Keywords:** surgical cancellation, preoperative, quality improvement, operating room, elective surgical procedures

## Abstract

Background

The operating room (OR) is a critical facility that consumes a significant percentage of the hospital's resources, so it must be used judiciously. Surgical cancellation is a chief cause of OR underutilization. The purpose of this study was to hold medical concerns accountable for surgical cancellations at a large tertiary care hospital in Maharashtra, India.

Methods

The Plan, Do, Study, and Act (PDSA) cycle is a tool for analyzing change and learning via action. We used this method to determine the origins of errors, identify key points, and test change interventions such as an individually tailored anesthetic plan, provision of a pre-anesthesia evaluation clinic in the outpatient department, reevaluating patients, and rechecking the preoperative checklist. This study was undertaken as a part of a quality improvement project at our hospital in India. All elective surgical operations scheduled between January and November 2020 were included, and canceled procedures were investigated to identify potential reasons.

Results

During the auditing period, 7,709 elective operations were scheduled; 68 (0.88%) of them were canceled. After piloting interventions, the rate of cancellations dropped from 1.08% to 0.67% in the succeeding cycle. A root cause analysis of the data revealed that there was a 7.1% decrease in cancellations due to hypertension, a 3.8% decrease due to insufficient routine blood tests, and a 1.9% decrease in the inappropriate preoperative workup, while we saw an increase in fever (5.5%) and blood sugar level (1.9%) discrepancies.

Conclusions

Dr. D. Y. Patil Hospital & Research Centre in Pune, India, had cancellations in scheduled ORs due to associated medical co-morbidity that were potentially reducible post-intervention and could be replicated for application in various tertiary care hospitals. Regular monthly audits, quality improvement projects, and the designation of an organized system may enhance the proper utilization of the OR which could potentially save funds, preserve resources, alleviate the burden of patients, and reduce cancellations to a minimum.

## Introduction

The operating room (OR) is the pivot of any tertiary hospital, requiring a considerable workforce and expenditure from the hospital budget. Canceling elective surgery on the scheduled date causes psychological anguish not only to the operating surgeons, OR technicians, ward nurses, and perioperative anesthetists but also prolongs and worsens patient suffering. These cancellations may result in disgruntled patients and may be costly, but they are potentially avoidable if proper administrative actions are taken [1,2].

Delayed cancellation of scheduled operations is a major cause of inefficient use of OR time and a misuse of resources. It is also potentially stressful, with demoralizing effects, and costly to the patient in terms of disruption of daily life and working days lost [3,4]. Cancellation of elective operations was a criterion to assess the quality of patient care and the management system. There are many reasons for the cancellation of elective surgical cases, and they differ within hospitals [5].

Our hospital has a designated quality team that oversees periodic continuous quality improvement (QI) initiatives. Although existing structured audit cycles are in place, there might be a gap in understanding of the present practice among different hospital staff members, such as nursing staff and surgeons.

This QI project was designed to investigate the prevalence of cancellations in elective surgery on the scheduled day and to find reasons for the failure of the same through the usage of a Plan, Do, Study, and Act (PDSA) cycle in our tertiary care hospital within 10 months.

## Materials and methods

We defined a surgical cancellation as the non-performance of a surgery that was scheduled for a particular date and time, due to medical reasons or acute exacerbation of existing co-morbidity.

All patients who required surgery at our hospital were first evaluated in the outpatient department and then hospitalized. They were examined in the pre-anesthetic evaluation clinic before surgery to obtain fitness clearance. The reason for this is due to our target population being rural and the unavailability of a Health Information System (HIS). We rely on handwritten notes and patients come in from interiors of the state as well as villages around the city hence it is logistically easier for PACs, referrals, and optimizations to be done for the admitted patients instead of having them go back and forth. We studied elective OR bookings and postponements in various surgical disciplines at a large tertiary charitable hospital in Maharashtra (2,000 resourced beds, 26 ORs) for 10 months. However, factors such as patient absenteeism, surgeon availability, the lack of or overuse of rooms during emergency procedures, and a lack of equipment were excluded from our analysis.

A pre-post interventional study was carried out, consisting of a control, and study period. During the first five months (January-June), data were meticulously collected, and it was hoped that by adopting modifications, refinements would be made in the coming months (July-November). All surgeries were canceled in April due to SARS-CoV-2 but were subsequently resumed after employing a rapid reverse transcription-polymerase chain reaction (RT-PCR) test prior to all admitted patients. We designed an audit tool in Microsoft Office Excel that was utilized for data collection, analysis, and chart generation and adhered to SQUIRE 2.0 guidelines for preparing the manuscript [6].

**Figure 1:**
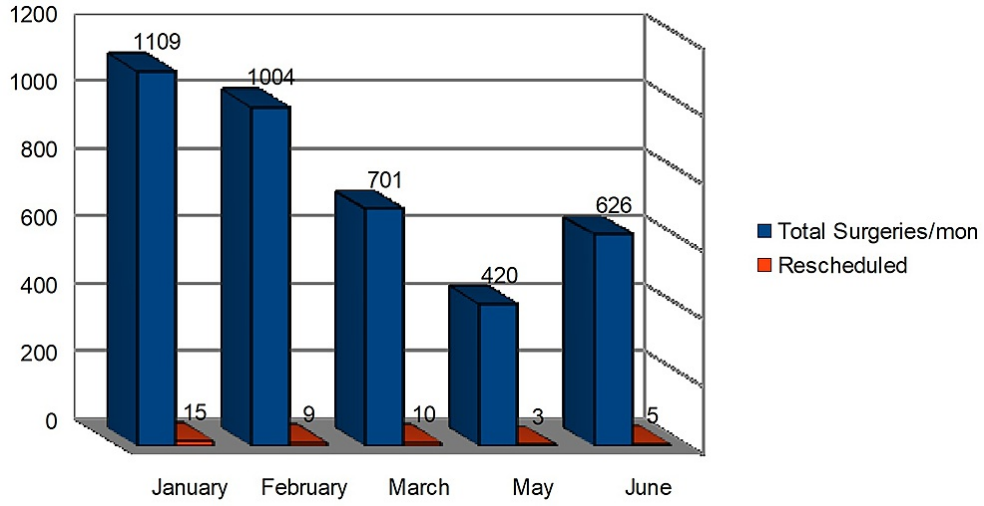
The control data collected from the month of January-June in the year 2020 comparing the total surgeries (n=3,860) and rescheduled (n=42) due to various predisposed medical comorbidity mon = Month

During retrospective data collection and analysis (control period), it was revealed that many on-the-day elective surgery cancellations were potentially avoidable. We found out that March had the highest number of rescheduled surgeries, while May had the least (Figure 1).

**Figure 2:**
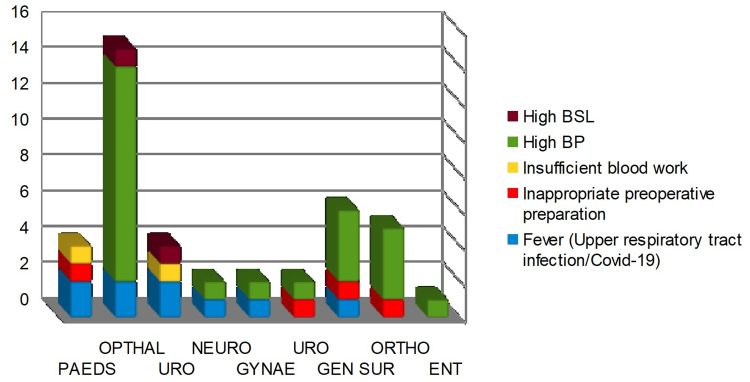
The control data collected from the month of January-June in the year 2020 comparing the breakdown of predisposed medical comorbidity in various surgical subspecialties BP = Blood pressure; BSL = Blood sugar level; Opthal = Ophthalmology; Neuro = Neurosurgery; Paeds = Pediatrics; Uro = Urology; Gen Sur = General Surgery; Gynae = Gynecology; Ortho = Orthopedics

A root cause analysis study into the data revealed that the most common medical issue was hypertension (57.1%), followed by fever caused by either upper respiratory tract infections or SARS-CoV-2 (21.4%), inappropriate preoperative preparation requiring further assessment (11.9%), insufficient routine blood tests (7.1%), and the uncommon one was high blood sugar (4.7%). Due to cancellations caused by hypertension, the Department of Ophthalmology was the most afflicted, followed by General Surgery and Orthopedics (Figure 2).

Our data showed that there was a requirement for a personally tailored plan for each patient to optimize the management of their disease and prevent delays. Some were due to preventable issues like hypertension (>160/100 mm Hg), insufficient routine blood tests, or inappropriate preoperative preparation, while others were unpredictable, like fever (>38° C/100.4° F) before the scheduled surgery.

Our objective was to create a plan that had the patient's best interests while being practical and improving the quality of care provided through continuous audit cycles. We tried to prevent perioperative medical complications by strengthening and implementing interdepartmental collaboration and customizing treatment approaches. This was accomplished by discussing flaws with the preoperative assessment team and then following up by piloting interventions.

Due to the ongoing SARS-CoV-2 pandemic, it was difficult to arrange a physical meeting. We, therefore, opted to put forward our concerns and findings during the monthly audit meeting on the online video conferencing platform, Zoom. The Dean, the quality team, the OR team, the nursing staff, and several departmental heads from surgical and medical subspecialties attended this meeting.

**Figure 3:**
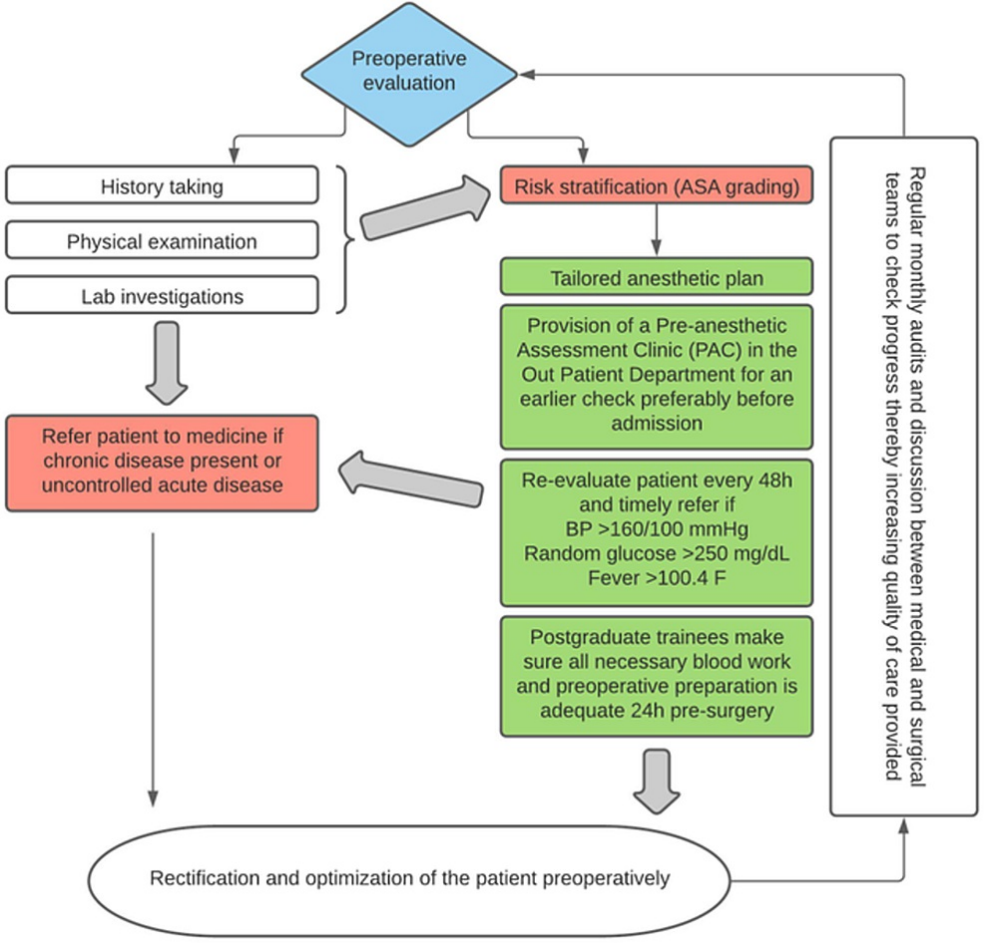
The collective intervention steps devised by our Quality Team to reduce the preoperative cancellations of scheduled cases ASA = American Society of Anesthesiology Physical Status Classification

All departments agreed that a customized strategy for each patient should be piloted, and pre-anesthetic evaluation should be performed sooner with the availability of an anesthesia clinic in the outpatient department. Vital signs such as blood pressure, blood sugar levels, temperature, and preoperative workup were monitored every 48 hours by resident doctors to reduce errors, and any disparities were immediately reported to the medical team (Figure 3).

It was emphasized that the anesthetic department should effectively coordinate with medical specialty departments to make prompt referrals of patients with preexisting medical comorbidity for whom suitable care should be determined according to local hospital protocols. To limit the amount of inaccuracy in data collection, the nursing staff were encouraged to electronically capture data using Microsoft Office Excel sheets (Figure 3).

## Results

Insufficient preoperative medical optimization is the cornerstone of our study, which is an important reason for the cancellation of scheduled cases. The most common causes were hypertension, respiratory tract infections caused by SARS-CoV-2/nosocomial infections, inadequate blood testing, and poor preoperative optimization (requiring further assessment prior to surgery). A day or two before surgery, the anesthetist at our institution evaluated several of the patients, and those who warranted preoperative medical optimization were referred to a physician.

**Figure 4:**
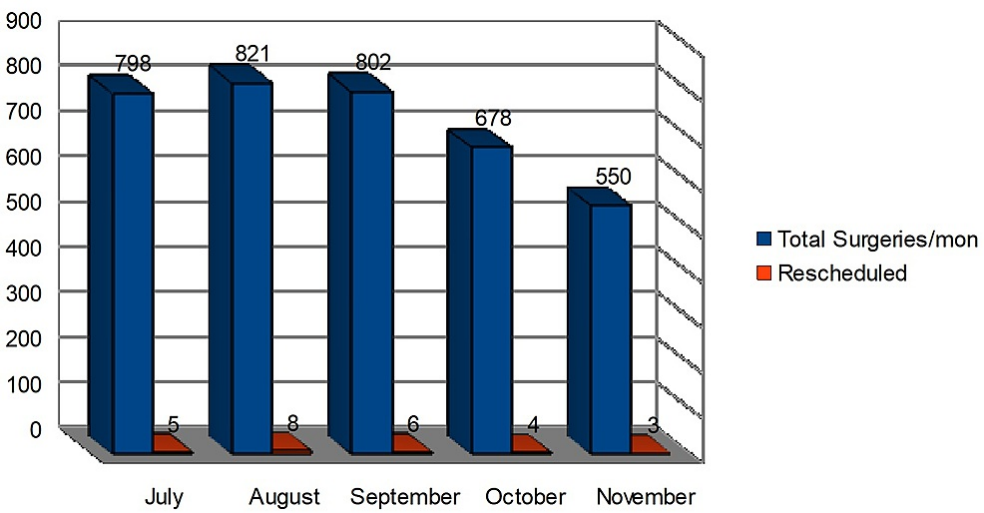
The study period data collected from the month of July-November in the year 2020 comparing the total surgeries (n=3,849) and rescheduled (n=26) due to various predisposed medical comorbidity mon = Month

After admitting shortcomings to the preoperative assessment team, we proceeded with a retrospective analysis of prospectively maintained data spanning July to November 2020. The trend of cancellations has slightly declined, with August having one of the highest and November having the fewest (Figure 4).

After piloting interventions, the rate of cancellations dropped from 1.08% to 0.67% in the succeeding cycle. A root cause analysis of the data implied that there was a decrease in cancellations due to hypertension by 7.1%, insufficient blood work by 3.8%, and inappropriate preoperative workup by 1.9%, while we saw an increased incidence of fever (5.5%) and blood sugar level (1.9%) discrepancies (Table 1).

**Table 1:** Summary of medical reasons for cancellation

Reason for cancellation (%)	Control period (%)	Study period (%)
Hypertension (>160/100)	57.1	50
Fever due to SARS-CoV-2/ nosocomial infections (>100.4 F)	21.4	26.9
Inappropriate preoperative preparation (requiring further management)	11.9	10
Insufficient blood work	7.1	3.3
Random blood sugar levels (>250 mg/dl)	4.7	6.6

**Figure 5:**
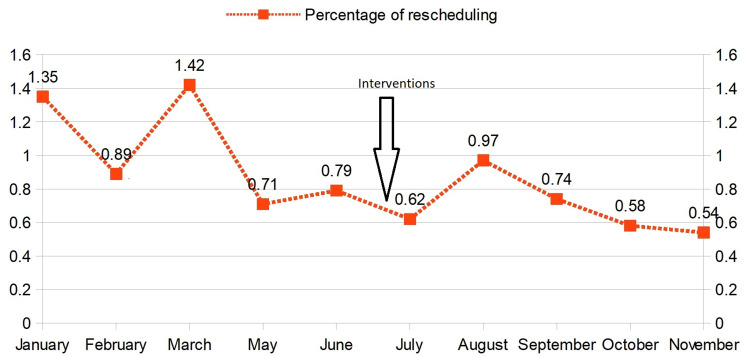
Comparing the trend of percentage (%) of rescheduled surgeries from the month of January-November in the year 2020

We plotted a line graph on the percentage (%) of rescheduled surgeries throughout the study and found a decline in the cases after the interventions took place. The most likely causes are enhanced communication among personnel and also across departments, as well as a definite structural design (Figure 5).

## Discussion

Piloting a preoperative assessment clinic in the outpatient department was feasible and optimized resource use as well as man-hours. Having the clinic embedded internally saves the patient a trip and optimizes referral for associated medical co-morbidity. Reevaluating patients every 48 hours and having a set value for medical referrals also brings down the rate of rescheduling.

Hussain et al. stated that anesthesia was the cause of 8% of the canceled surgeries [7]. Yet most surgeons schedule their patients for surgery straight away after physician reference without optimizing the state of the patient. This trend was changed by having an anesthesia clinic in the outpatient department itself. Studies have shown that preoperative anesthesia evaluation in pre-anesthetic evaluation clinics notably reduces operative room setbacks and cancellations [8,9].

Defined procedures may necessitate not just modifications among surgeons, anesthesiologists, and nurses, but also the planning, scheduling, and assembly of expensive specialized equipment. Interns, technicians, and medical students may also be required to participate in surgeries. As a result, surgical cancellations result in a substantial waste of time, energy, and both human and financial resources [9].

At an individual level, patients may have to change their schedules to accommodate a surgery date, travel a long distance, take unpaid leave from work, and/or arrange for childcare. Moreover, patients may be directed to modify or abstain from medication regimens before surgery. The aftermath of surgical cancellations thus extends beyond the exploitation of financial resources for the hospital [8,9].

From a QI viewpoint, low surgical cancellation rates are an equitable indicator of the efficient use of an OR as well as the hospital’s monetary resources. In a substantial study comprised of 329,784 surgical cases booked across nine different surgical specialties at 40 hospitals in the U.S.A., the overall surgical cancellation rate was 12.4%; medical bases were accountable for 28% of cancellations [10].

In the current study, 21.4% of cancellations were due to acute conditions such as fever. A similar rate of upper respiratory tract infection-related cancellations was described by Rodríguez et al., although this decreased from 12.9% in 2001 to 7.2% in 2003 through enhanced communication of uncomplicated and apt information to parents [11]. However, a few components in our study might be affected due to the ongoing pandemic, which might have led to altered results.

In the opinion of the study by Ferschl et al., the recurrent causes for canceling elective cases on the day of surgery were: inadequate preoperative preparation (21%), upper respiratory tract infection (19%), and hypertension (13%) [12]. Knox et al. detailed a noteworthy reduction in medical-related surgical cancellations after establishing a preoperative assessment clinic (P = 0.013) [13].

However, a few impediments delayed our findings. Since the nursing staff collected data using non-electronic methods, there may have been some mistakes during data acquisition and transmission onto a computer. A monthly meeting with the whole hospital staff was difficult because everyone had responsibilities. Some concerns were inevitable, such as cancellations caused by the ongoing SARS-CoV-2 infection, which might result in increased postoperative morbidity and death.

Medical issues can be discovered in time and the frequency of cancellations on medical grounds is reduced by establishing a formal liaison with physicians and improving communication between nurses, doctors, and patients. The results from this study may serve to increase awareness of the burden of surgical cancellation due to underlying medical issues and encourage similar audits at other hospitals in India.

## Conclusions

Dr. D. Y. Patil Hospital & Research Centre had cancellations due to associated medical co-morbidity that was potentially reducible post-intervention and could be replicated for application in various tertiary care hospitals. Regular monthly audits, QI projects, and the designation of an organized system may enhance the proper utilization of the OR, which could potentially save funds, preserve resources, alleviate the burden of patients, and reduce cancellations to a minimum.
